# Circulating GDF15 and HbA1c Response to Add-On Exenatide Therapy in Type 2 Diabetes: A Post Hoc Analysis from a Multicenter Trial

**DOI:** 10.3390/biomedicines14030572

**Published:** 2026-03-03

**Authors:** Qi Wu, Kun Yang, Xinyue Liao, Shiyin Zheng, Xiaoyue Zheng, Haining Wang, Jin Yang, Tianpei Hong

**Affiliations:** Department of Endocrinology and Metabolism, NHC Key Laboratory of Cardiovascular Molecular Biology and Regulatory Peptides, Peking University Third Hospital, Beijing 100191, China

**Keywords:** type 2 diabetes mellitus, growth differentiation factor 15, glucagon-like peptide-1 agonists, glycated hemoglobin

## Abstract

**Objectives**: To assess the influences of growth differentiation factor 15 (GDF15) on the reduction in glycated hemoglobin (ΔHbA1c) induced by exenatide in type 2 diabetes mellitus (T2DM). **Methods**: This analysis included 166 participants with T2DM who received exenatide as add-on therapy for 16 weeks. The effect of baseline GDF15 on ΔHbA1c was evaluated using univariate, multivariate, and bidirectional stepwise linear regression models. Baseline GDF15 was categorized into tertiles with the lowest tertile (tertile 1) serving as reference. A subgroup analysis was performed in the participants aged >35 years to investigate whether age influenced the effect of GDF15 on ΔHbA1c. **Results**: GDF15 levels were significantly increased from baseline following 16 weeks of exenatide treatment [721.9 (513.6, 997.8) pg/mL vs. 741.4 (510.0, 1203.4) pg/mL, *p* = 0.031]. Univariate linear regression analysis revealed a positive association between baseline GDF15 (tertile 3: *β* = 0.553, 95% CI 0.115 to 0.991, *p* = 0.014) and ΔHbA1c. However, no significant relationship was found between GDF15 (tertile 2: *p* = 0.403; tertile 3: *p* = 0.217) and ΔHbA1c after adjusting for age and diabetes duration. Further stepwise regression analysis indicated a non-robust association for GDF15 in the absence of age as GDF15 was excluded from the model. Among the participants >35 years old, GDF15 (tertile 3: *β* = 0.383, 95% CI 0.002 to 0.764, *p* = 0.049) remained positively associated with ΔHbA1c, even after adjusting for age. **Conclusions**: Elevated GDF15 might potentially diminish the reduction in HbA1c following 16-week exenatide treatment, with this effect moderated by age.

## 1. Introduction

Diabetes has become an important health issue worldwide, especially in China. The prevalence of diabetes continues to increase, having risen from 10.9% in 2013 to 12.4% in 2018–2019 in China [1,2]. In 2024, it is estimated that 148.0 million people have diabetes in Chinese adults, and this number is projected to reach 168.3 million by 2050 [3]. Poor glycemic control is prevalent in type 2 diabetes mellitus (T2DM), failing to meet the World Health Organization (WHO) objective that 80% of diagnosed diabetes patients achieve optimal glycemic management [4]. Thus, precise blood glucose control is an urgent goal that needs to be addressed.

Metformin remains the standard of monotherapy for T2DM, since it is favored for its global accessibility, efficacy, safety, and cost-effectiveness. In T2DM patients with atherosclerotic cardiovascular disease or high cardiovascular risk, glucagon-like peptide-1 receptor agonists (GLP-1RAs) were recommended as an important option [5]. Moreover, GLP-1RAs could benefit T2DM patients by reducing caloric intake and body weight, together with enhancing glucose-dependent insulin secretion, suppressing glucagon secretion, and slowing gastric emptying [6].

In addition to their effects on glycemic control, either GLP-1RAs or metformin can also regulate the production of cytokines and chemokines [7]. Our previous study showed that serum fibroblast growth factor 21 (FGF21) levels were elevated in T2DM patients after the GLP-1RA exenatide treatment, and FGF21 mediated the hepatic glucose output inhibition function of GLP-1RA [8]. Moreover, recent studies found that metformin elevated the levels of circulating growth differentiation factor 15 (GDF15), which was crucial for exerting its benefits on energy balance and body weight [9], as well as achieving full AMPK activation, the primary mechanism mediating its antidiabetic effects [10].

GDF15, also known as macrophage inhibitory cytokine 1, belongs to the transforming growth factor-β superfamily [11]. GDF15, which is induced by macrophage activation, has emerged as a stress factor closely linked to metabolic dysfunction, including obesity, cardiovascular disease, metabolic dysfunction-associated steatotic liver disease, and T2DM [12,13,14,15,16]. However, the role of GDF15 in the hypoglycemic function of GLP-1RAs has not been fully explored.

Therefore, this study aimed to investigate changes in GDF15 levels in T2DM patients treated with exenatide for 16 weeks, and whether baseline GDF15 levels influenced the magnitude of HbA1c reduction following exenatide treatment.

## 2. Materials and Methods

### 2.1. Study Design

The multicenter interventional trial was conducted at seven tertiary hospitals in China and registered with the Chinese Clinical Trial Registry (www.chictr.org.cn; number: ChiCTR-IPR-15006558; date: 27 May 2015). The Medical Ethics Committee of Peking University Third Hospital approved the study protocol (Approval Code: 11-69-II-NFM; date: 14 September 2011). Written informed consent was obtained from all participants before enrollment.

### 2.2. Participants

**Figure 1:**
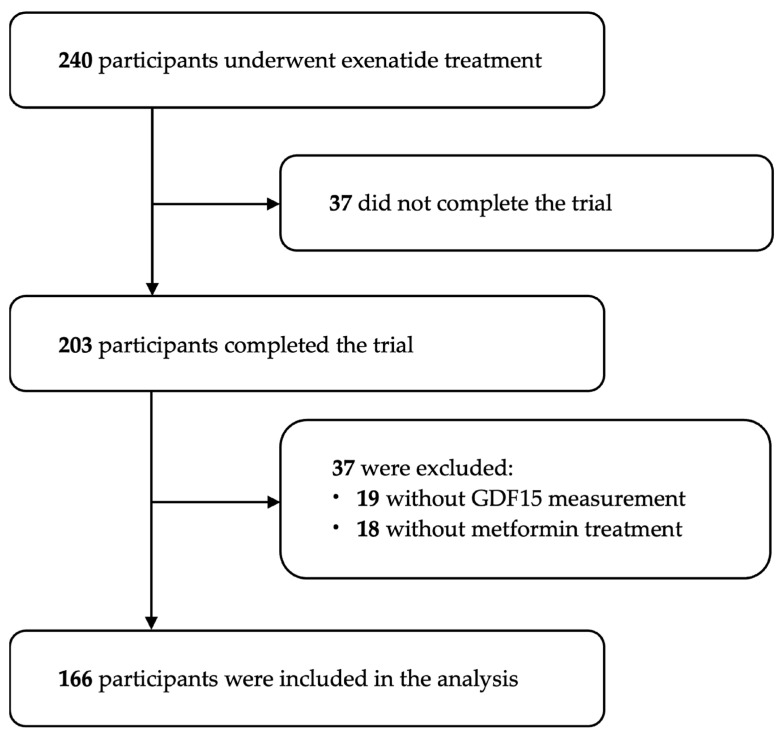
The flowchart of this study. Abbreviation: GDF15, growth differentiation factor 15.

The detailed inclusion and exclusion criteria for this trial have been reported elsewhere [17]. Briefly, eligible adults with T2DM (1999 WHO definition) aged 20~70 years, who were treated with metformin and insulin secretagogues alone or in combination, received subcutaneous exenatide for 16 weeks. Exenatide was initiated at 5 μg twice daily for the first 4 weeks and then increased to 10 μg twice daily for the subsequent 12 weeks (or continued the lower dose if tolerance issues arose). Between July 2011 and June 2013, 240 patients were randomly enrolled, of whom 203 completed the intervention. Nineteen individuals with incomplete GDF15 data at baseline or follow-up were excluded, and 18 participants without a background metformin therapy were also excluded, given the influence of metformin on GDF15 levels [9]. Ultimately, 166 individuals were included in this post hoc secondary analysis (Figure 1).

### 2.3. Anthropometric Measurement and Biochemical Examination

Anthropometric measurement and biochemical examination of participants at baseline and post-treatment time points were collected. The term “baseline” refers to the screening period when patients had not yet started exenatide treatment, and “post-treatment” refers to the follow-up period at the end of the 16-week exenatide treatment. Information on age, gender, diabetes duration, and background therapies (metformin, insulin secretagogues) was obtained from medical records. Height and weight were measured without shoes and outerwear. Body mass index (BMI) was calculated as weight in kilograms divided by height in meters squared (kg/m^2^). Systolic and diastolic blood pressures were measured three times in the same arm after at least 5 min of rest on each occasion, and the mean value was utilized for analysis. Fasting blood glucose, 2 h postprandial glucose, glycated hemoglobin (HbA1c), serum triglycerides, total cholesterol, low-density lipoprotein cholesterol (LDL-C), high-density lipoprotein cholesterol (HDL-C), alanine aminotransferase, aspartate aminotransferase, alkaline phosphatase, total bilirubin, blood urea nitrogen, serum creatinine, uric acid, amylase, and lipase were measured. The post-treatment HbA1c level minus its baseline level yields the reduction from baseline in HbA1c level (ΔHbA1c). The fasting leftover serum at baseline and post-treatment were collected and stored in −80 °C. The serum GDF15 levels were detected by using a human GDF15 enzyme-linked immunosorbent assay kit (Cat: DGD150, R&D Systems, Minneapolis, MN, USA). The detection of GDF15 was performed on the same day by the same operator.

### 2.4. Statistical Analysis

Continuous variables with normal distributions are described as mean ± standard deviation, while data that were not normally distributed are presented as median (interquartile range). The differences between baseline and post-treatment parameters were examined using a paired *t*-test or a Wilcoxon test. The correlation between the two variables was illustrated using scatter plots. Correlation analyses were conducted using Spearman’s rank method. In cases where ties were present, Kendall’s *tau* correlation coefficient was employed to assess the correlation [18]. Univariate and multivariate linear regression analyses were performed to examine the association between baseline variables and ΔHbA1c. Considering the relatively small sample size in this study, we further performed a bidirectional stepwise regression analysis to simplify the model and identify the strongly correlated variables. The stepping criteria employed for entry and removal were based on the significance level of the *F*-value and set at 0.05 [19]. Stepwise regression analysis was employed solely as an exploratory tool to understand potentially important variables. The coefficient *β* and its 95% confidence interval (CI) were reported. Baseline GDF15 levels were categorized into tertiles in the model, corresponding to the 33rd and 67th percentiles with the lowest tertile (tertile 1) serving as reference. All statistical analyses were performed using R software (version 4.3.3). The plots were generated using the forestploter (version 1.1.3), ggplot2 (version 4.0.1) and ggpubr (version 0.6.1) packages. *p* < 0.05 was considered significant.

## 3. Results

### 3.1. Characteristics of the Participants at Baseline and After the Exenatide Treatment

The comparison of baseline characteristics between the 166 included participants and 37 excluded participants is shown in Table S1. Most parameters were similar between the two groups, indicating that the selection bias might not affect the results of this post hoc analysis.

The anthropometric and biochemical characteristics of the study participants at baseline and post-treatment are shown in Table 1. The average age of the participants was 49.8 ± 10.0 years, and the median diabetes duration was 4.79 years (interquartile range 2.33 to 8.58). Among 166 participants, 101 were male, accounting for 60.8%. In addition to the significant improvements in glucose metabolism parameters, including fasting blood glucose, 2 h postprandial glucose and HbA1c (all *p* < 0.001), weight, BMI, serum HDL-C and total bilirubin levels were also significantly decreased after 16 weeks of exenatide treatment (all *p* < 0.05). In contrast, serum amylase and lipase levels were remarkably increased after the exenatide treatment (both *p* < 0.001). Two participants had serum amylase values slightly above the laboratory upper limit of normal (ULN) of 135 U/L (an increase of less than 13.2 U/L), and four participants exhibited lipase values marginally above the ULN of 300 U/L (an increase of less than 50 U/L). However, none reported the related abdominal symptoms, and no participants met the clinical diagnostic criteria for pancreatitis. It was notable that GDF15 levels were markedly elevated after the exenatide treatment (721.9 [513.6, 997.8] pg/mL vs. 741.4 [510.0, 1203.4] pg/mL, *p* = 0.032). Moreover, the Kendall’s test showed no significant associations between the change in GDF15 and change in serum amylase (*tau* = 0.051, *p* = 0.334; Figure S1A) or lipase (*tau* = 0.056, *p* = 0.289; Figure S1B). Thus, the safety of exenatide treatment was acceptable.

### 3.2. Baseline GDF15 Is Positively Associated with ΔHbA1c in Univariate Linear Regression Analysis

To better capture effects of baseline GDF15 and improve the robustness of our analysis, we categorized baseline GDF15 into tertiles for inclusion in the model, and the baseline characteristics are shown in Table 2. Age (*p* = 0.002) and diabetes duration (*p* = 0.001) increased with higher baseline GDF15 tertiles. The mean baseline HDL-C levels rose to 1.31 mmol/L in the second tertile before declining to 1.20 mmol/L in the highest tertile versus 1.26 mmol/L in the lowest tertile (*p* = 0.047). Although the baseline HbA1c levels did not differ significantly among the tertile groups, smaller reduction from baseline in HbA1c levels as indicated by higher ΔHbA1c was found in higher baseline GDF15 tertiles (*p* = 0.046).

The univariate linear regression analysis of ΔHbA1c and baseline variables is shown in Table 3. Age (*β* = 0.032, 95% CI 0.015 to 0.049, *p* < 0.001), total cholesterol (*β* = 0.243, 95% CI 0.062 to 0.424, *p* = 0.009), LDL-C (*β* = 0.246, 95% CI 0.044 to 0.447, *p* = 0.017), and third tertile of baseline GDF15 (*β* = 0.553, 95% CI 0.115 to 0.991, *p* = 0.014) were positively associated with ΔHbA1c, whereas baseline HbA1c showed a negative association (*β* = −0.679, 95% CI −0.846 to −0.512, *p* < 0.001). The diabetes duration (*β* = 0.033, 95% CI −0.004 to 0.070, *p* = 0.079) and systolic blood pressure (*β* = 0.014, 95% CI −0.0003 to 0.028, *p* = 0.056) were in borderline association with ΔHbA1c. Considering the strong correlation between baseline total cholesterol and LDL-C (*ρ* = 0.879, *p* < 0.001; Figure S2), only total cholesterol was included in the subsequent multivariate linear regression model, as it exhibited a smaller *p*-value.

### 3.3. Multivariate and Bidirectional Stepwise Linear Regression Analyses of ΔHbA1c and Baseline Variables

**Figure 2:**
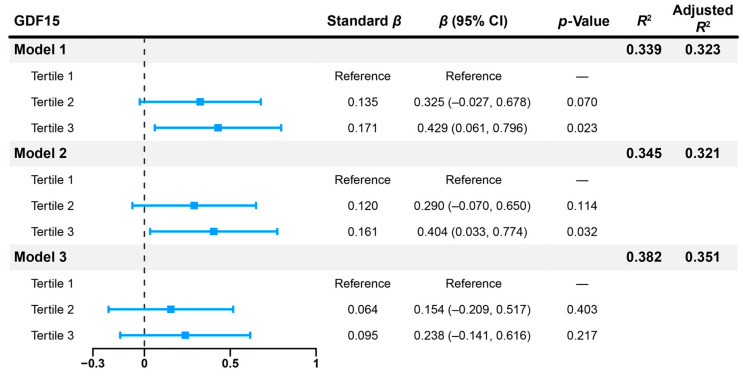
Forest plot for multivariate regression analysis of ΔHbA1c with baseline GDF15 tertiles adjusted for different baseline variables. Model 1 was adjusted for baseline HbA1c and total cholesterol. Model 2 was adjusted for baseline HbA1c, total cholesterol, systolic blood pressure, and BMI. Model 3 was adjusted for age, diabetes duration, baseline HbA1c, total cholesterol, systolic blood pressure, and BMI. Δ means the post-treatment level minus its baseline level. Abbreviations: HbA1c, glycated hemoglobin; GDF15, growth differentiation factor 15; BMI, body mass index.

As shown in Figure 2, after adjusting for baseline HbA1c, total cholesterol, systolic blood pressure, and BMI, third tertile of baseline GDF15 (Model 2: *β* = 0.404, 95% CI 0.033 to 0.774, *p* = 0.032) remained positively correlated with ΔHbA1c. However, when age and diabetes duration were included in the model, third tertile of baseline GDF15 (Model 3: *β* = 0.238, 95% CI −0.141 to 0.616, *p* = 0.217) was no longer associated with ΔHbA1c. In a further bidirectional stepwise regression analysis, baseline GDF15 was excluded from the model, and age (*β* = 0.018, 95% CI 0.002 to 0.035, *p* = 0.024), total cholesterol (*β* = 0.205, 95% CI 0.056 to 0.354, *p* = 0.007), and baseline HbA1c (*β* = −0.658, 95% CI −0.819 to −0.496, *p* < 0.001) were the most important factors influencing ΔHbA1c (Table S2).

### 3.4. Multivariate and Bidirectional Stepwise Linear Regression Analyses of ΔHbA1c and Baseline Variables Among the Participants >35 Years Old

Age may act as a confounding factor influencing the effect of baseline GDF15 on ΔHbA1c. According to the “Youth in the Medium- and Long-term Youth Development Plan (2016–2025)” in China [20], 35 years is the cut-off for classifying younger and older adults. The baseline characteristics of both groups are presented in Table S3. Higher age and longer diabetes duration, and higher baseline GDF15 levels were observed in the participants >35 years old. In the participants aged >35 years (*n* = 148), baseline GDF15 exhibited a borderline positive correlation with ΔHbA1c (*ρ* = 0.139, *p* = 0.093; Figure S3A), whereas in the participants aged ≤35 years (*n* = 18), baseline GDF15 appeared to be negatively correlated with ΔHbA1c (*ρ* = −0.448, *p* = 0.062; Figure S3B).

**Figure 3:**
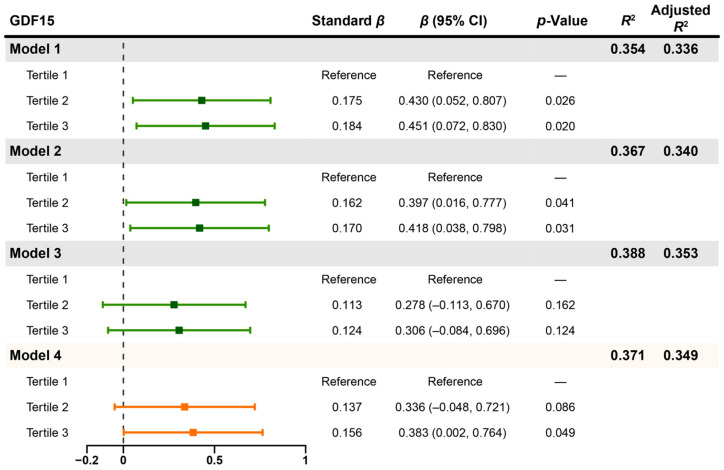
Forest plot for multivariate regression analysis of ΔHbA1c with baseline GDF15 tertiles adjusted for different baseline variables. Model 1 was adjusted for baseline HbA1c and total cholesterol. Model 2 was adjusted for baseline HbA1c, total cholesterol, systolic blood pressure, and BMI. Model 3 was adjusted for age, diabetes duration, baseline HbA1c, total cholesterol, systolic blood pressure, and BMI. Model 4 was bidirectional stepwise regression analysis for Model 3. Δ means the post-treatment level minus its baseline level. Abbreviations: HbA1c, glycated hemoglobin; GDF15, growth differentiation factor 15; BMI, body mass index.

We conducted a univariate linear regression analysis of ΔHbA1c in the participants aged >35 years. Similar to the findings from the overall participants, age (*p* = 0.004), systolic blood pressure (*p* = 0.037), total cholesterol (*p* = 0.004), LDL-C (*p* = 0.009), baseline HbA1c (*p* < 0.001), and third tertile of baseline GDF15 (*p* = 0.012) were significantly associated with ΔHbA1c (Table S4). After adjusting for baseline HbA1c, total cholesterol, systolic blood pressure, and BMI, both second tertile (Model 2: *β* = 0.397, 95% CI 0.016 to 0.777, *p* = 0.041) and third tertile (Model 2: *β* = 0.418, 95% CI 0.038 to 0.798, *p* = 0.031) of baseline GDF15 remained significantly correlated with ΔHbA1c (Figure 3). However, this correlation disappeared after further adjustment for age and diabetes duration (Model 3; Figure 3). Moreover, subsequent stepwise regression analysis identified baseline GDF15, age, total cholesterol, and baseline HbA1c as influencing factors. Notably, third tertile of baseline GDF15 remained positively associated with ΔHbA1c, even after adjusting for age (Model 4: *β* = 0.383, 95% CI 0.002 to 0.764, *p* = 0.049; Figure 3). The detailed results of stepwise regression analysis are provided in Table S5. Thus, although age might be a cofounding factor obscuring the effects of GDF15, baseline GDF15 continues to be a potential factor positively associated with the magnitude of HbA1c reduction in T2DM participants >35 years old.

For the participants ≤35 years old, since the sample size is quite small, continuous GDF15 level, instead of tertile-based stratification, was exploited in the linear regression model. To avoid overfitting due to the small sample size, the multivariable model was restricted to baseline GDF15 and baseline HbA1c, and neither of these two variables was significantly associated with ΔHbA1c (GDF15: *p* = 0.264, and HbA1c: *p* = 0.067; Table S6).

## 4. Discussion

Our post hoc secondary analysis found that circulating GDF15 levels were significantly increased after 16 weeks of the GLP-1RA exenatide treatment in T2DM patients who were treated with metformin alone or in combination with insulin secretagogues. Although the baseline HbA1c levels were not significantly different among the baseline GDF15 tertiles, smaller reduction from baseline in HbA1c levels was observed in higher baseline GDF15 tertiles. Furthermore, multivariate linear regression analysis indicated that age and diabetes duration moderated the effect of baseline GDF15 on HbA1c reduction. The age-stratified subgroup analysis showed that among the participants >35 years old, baseline GDF15 remained a positive association with HbA1c reduction, even after adjusting for age. By contrast, the participants ≤35 years old appeared to exhibit a negative relationship between baseline GDF15 and HbA1c reduction. Therefore, age might be an important factor influencing the effect of GDF15 on HbA1c reduction, albeit baseline GDF15 continued to be a potential factor positively associated with HbA1c reduction in T2DM participants >35 years old. However, these age-specific observations are exploratory and require further confirmation in larger cohorts.

Studies showed that GLP-1RAs could reduce the levels of pro-inflammatory cytokines and chemokines [7,21]. Our previous study found that FGF21, a liver-derived cytokine, was elevated after a 16-week exenatide treatment [8]. In this study, we revealed that GDF15 was also increased after a 16-week exenatide treatment, even in the background of metformin usage. Unfortunately, due to the absence of a non-exenatide control group, we could not establish a causal link between GLP-1RA use and increased circulating GDF15 levels. According to our knowledge, there are currently no studies reporting the impact of exenatide alone on GDF15 levels. By contrast, other studies indicated no relationship between GLP-1RA treatment and circulating GDF15 levels. Valenzuela-Vallejo et al. [22] found that the levels of total and intact GDF15 remained unchanged after 5 weeks of liraglutide treatment. For a 180-day liraglutide treatment, GDF15 levels also remained relatively stable among patients with heart failure [23] as well as individuals with overweight or obesity [24]. Possible reasons for those findings that differed from ours might include differences in the types of GLP-1RA or in disease status. In addition, GLP-1RA was an add-on therapy to the background therapy of metformin alone or in combination with insulin secretagogues in our study, which may also have an impact on the GDF15 levels.

Circulating GDF15 levels are dynamically regulated and increased with age, pregnancy, smoking, physical exercise, adiposity, and various disease states, as well as metformin intervention [12]. It has been shown that individuals with higher GDF15 levels tend to have worse metabolic conditions. Cohort studies found that GDF15 levels were significantly elevated in individuals with impaired glucose tolerance [25,26] and T2DM [27]. Furthermore, multi-omics studies revealed that the circulating GDF15 level was significantly associated with a higher risk of T2DM development and progression [28,29]. In line with the findings from previous studies, our study indicated that higher baseline GDF15 levels are associated with a poorer metabolic condition, as evidenced by a smaller reduction in HbA1c levels among the individuals with higher GDF15 tertiles. However, our results also found that among T2DM patients ≤35 years old, baseline GDF15 appeared to be negatively associated with HbA1c reduction. To the best of our knowledge, our study is the first to investigate the association between baseline GDF15 and HbA1c reduction in the GLP-1RA-treated T2DM patients. Consequently, these results imply that higher GDF15 levels are linked to a poorer metabolic condition and a less favorable response to exenatide in T2DM patients aged >35 years.

Notably, the relationship between baseline GDF15 and HbA1c reduction was influenced by age and diabetes duration in our study. Compared to first tertile group of baseline GDF15, second and third tertile groups whose average ages were over 50 years old showed a significant positive association with both older age and longer diabetes duration. A previous study indicated that GDF15 levels increased with advancing age, exhibiting a more rapid rise in individuals over the age of 50 [30]. This observation suggests that elevated GDF15 levels may, in part, stem from increasing age, and that age along with diabetes duration exerts a more pronounced effect on HbA1c reduction. Given the relatively small sample size in our study, future studies are warranted to validate the associations of GDF15 level with HbA1c reduction, and to clarify the role of age in this relationship.

Recently, it has been demonstrated that GDF15 may also hold potential as a therapeutic target for T2DM. Zhang et al. [31] found that elevated GDF15 levels in prediabetes and T2DM individuals were positively correlated with increased C-peptide levels, implying a compensatory mechanism by which GDF15 might support β-cell function under metabolic stress. Metformin is known to exert weight loss and appetite inhibition effects by elevating GDF15 levels [9,32]. Moreover, exogenous administration of long-acting GDF15 molecules could reduce food intake and body weight, and improve glycemic and lipid metabolism parameters [33]. Mechanisms of the pancreatic β-cell protection by GDF15 might include activating the canonical insulin secretion signaling pathway [31], increasing macrophage oxidative function and driving it toward an M2-like phenotype [34], and protecting against glucotoxicity-mediated defects in glucose-stimulated insulin secretion and β-cell viability [35].

The apparent contradiction regarding the role of GDF15 may arise because physiological or slight elevations in its circulating concentration, in the context of metabolic dysfunction, are insufficient to exert a therapeutic benefit. Unlike other cytokines, GDF15 is present at relatively high concentrations, ranging from approximately 200 to 1200 pg/mL in circulation [36]. A previous study indicated that the circulating GDF15 level was elevated to about 300 pg/mL in high-fat diet-induced obese mice, a modest three-fold rise compared to lean controls, while therapeutic efficacy for appetite suppression required peak plasma levels of recombinant GDF15 around 50,000 pg/mL [37]. Engelbeen et al. [38] also found that the physiological loss of endogenous GDF15 and FGF21 has little impact on weight regulation or glycemic homeostasis in mice. By contrast, the pharmacological dosage of long-acting GDF15 analogs could achieve a significant 8~10% weight loss in obese mice. Thus, the moderate elevation of GDF15 levels in people with obesity and T2DM might remain a compensatory response that attempts to counteract the metabolic dysfunction. In line with those findings, the GDF15 levels observed in our study remained within a physiological range. This suggested that the GDF15 elevation might serve primarily as a compensatory mechanism reflecting the advancing age and adverse metabolic outcomes, compromising the therapeutic effect of exenatide on HbA1c reduction.

This secondary analysis is based on data from a multicenter study, suggesting that our results are somewhat robust. Apart from the elevation of GDF15 levels, which represented a metabolic dysfunction reported by previous studies [27,28,29], our analysis also indicated that higher baseline GDF15 levels were associated with a smaller HbA1c-lowering effect of exenatide. Several limitations of our analysis should be acknowledged. First, all participants in our study are Chinese, making the findings hard to be generalized to a wide-range population. Second, the lack of non-exenatide controls limits this study to investigate the causal relationship between GLP-1RA administration and circulating GDF15 levels. Additionally, the influence of metformin on GDF15 levels further restricts the ability to assess the association between exenatide treatment and GDF15 levels. Third, the sample size of this study was relatively small, and the exclusion of 37 participants further reduced the sample size and might introduce selective bias. In addition, the lack of analysis of participants with background therapy of insulin secretagogue alone would also introduce treatment-related confounding. Therefore, whether elevated GDF15 levels compromise the hypoglycemic effect of GLP-1RA treatments requires verification in larger cohorts, particularly through prospective studies to investigate the association between GDF15 levels and therapeutic effect parameters.

## 5. Conclusions

Our study shows that circulating GDF15 levels are significantly increased in T2DM patients who are treated for 16 weeks with the GLP-1RA exenatide as an add-on therapy to metformin alone or in combination with insulin secretagogues. Importantly, higher baseline GDF15 levels are associated with a smaller reduction in HbA1c, potentially influenced by age. Therefore, our study provides a new perspective wherein T2DM patients with lower GDF15 levels may respond more favorably to GLP-1RA treatment.

## Figures and Tables

**Table 1:** Characteristics of T2DM patients at baseline and post-treatment (*n* = 166).

Variables	Baseline	Post-Treatment	*p*-Value
Age, year	49.8 ± 10.0	—	—
Sex—male, *n* (%)	101 (60.8%)	—	—
Diabetes duration, year	4.79 (2.33, 8.58)	—	—
Weight, kg	80.3 ± 14.7	78.3 ± 14.5	<0.001
Body mass index, kg/m^2^	28.6 ± 4.14	27.8 ± 4.03	<0.001
Heart rate, bpm	76.5 ± 7.90	77.5 ± 8.23	0.110
Systolic blood pressure, mmHg	126.1 ± 12.6	126.0 ± 11.6	0.814
Diastolic blood pressure, mmHg	78.8 ± 8.24	77.5 ± 7.31	0.058
Fasting blood glucose, mmol/L	9.29 ± 1.77	8.00 ± 2.05	<0.001
2 h postprandial glucose, mmol/L	16.3 ± 3.34	13.2 ± 4.01	<0.001
HbA1c, %	8.23 ± 0.91	7.00 ± 1.02	<0.001
Triglycerides, mmol/L	1.67 (1.21, 2.77)	1.62 (1.23, 2.64)	0.455
Total cholesterol, mmol/L	4.88 ± 0.97	4.80 ± 0.92	0.456
LDL-C, mmol/L	2.95 ± 0.87	2.92 ± 0.84	0.536
HDL-C, mmol/L	1.26 ± 0.39	1.22 ± 0.33	0.034
Alanine aminotransferase, U/L	25.6 (17.4, 40.5)	24.2 (17.3, 36.0)	0.141
Aspartate aminotransferase, U/L	24.7 (18.0, 32.2)	23.1 (18.0, 30.0)	0.327
Alkaline phosphatase, U/L	68.3 (57.0, 81.0)	66.5 (56.0, 82.0)	0.059
Total bilirubin, μmol/L	13.0 ± 5.22	12.2 ± 5.26	0.003
Blood urea nitrogen, mmol/L	4.92 ± 1.32	4.85 ± 1.34	0.536
Serum creatinine, μmol/L	64.5 ± 14.5	65.8 ± 14.8	0.067
Uric acid, μmol/L	324.4 ± 86.4	329.4 ± 87.3	0.147
Serum amylase, U/L	51.1 (43.0, 62.0)	56.8 (47.0, 72.9)	<0.001
Serum lipase, U/L	64.0 (37.1, 100.8)	77.0 (42.4, 141.0)	<0.001
GDF15, pg/mL	721.9 (513.6, 997.8)	741.4 (510.0, 1203.4)	0.031

Data are presented as mean ± standard deviation, median (interquartile range), or *n* (%). Abbreviations: T2DM, type 2 diabetes mellitus; HbA1c, glycated hemoglobin; LDL-C, low-density lipoprotein cholesterol; HDL-C, high-density lipoprotein cholesterol; GDF15, growth differentiation factor 15.

**Table 2:** Baseline characteristics and ΔHbA1c according to the baseline GDF15 tertiles.

Variables	Tertile 1 (*n* = 56)	Tertile 2 (*n* = 59)	Tertile 3 (*n* = 51)	*p*-Value
GDF15, pg/mL	463.2 (386.0, 513.6)	738.1 (647.8, 791.8)	1341.9 (1010.5, 1477.0)	<0.001
Age, year	46.0 ± 9.75	51.4 ± 10.5	52.1 ± 8.74	0.002
Sex—male, *n* (%)	33 (58.9%)	41 (69.5%)	27 (52.9%)	0.195
Diabetes duration, year	3.17 (2.06, 6.12)	5.08 (3.12, 10.1)	6.25 (3.38, 10.3)	0.001
Weight, kg	81.3 ± 13.7	80.4 ± 16.6	79.2 ± 13.8	0.777
Body mass index, kg/m^2^	28.8 ± 4.10	28.2 ± 4.20	28.8 ± 4.14	0.550
Heart rate, bpm	75.2 ± 8.40	78.7 ± 8.21	75.6 ± 6.47	0.051
Systolic blood pressure, mmHg	123.1 ± 12.4	128.1 ± 12.9	127.1 ± 11.9	0.130
Diastolic blood pressure, mmHg	78.2 ± 8.83	79.5 ± 8.74	78.7 ± 6.97	0.713
Fasting blood glucose, mmol/L	9.38 ± 1.94	9.29 ± 1.80	9.19 ± 1.57	0.997
2 h postprandial glucose, mmol/L	15.5 ± 3.84	16.6 ± 3.19	16.7 ± 2.79	0.071
HbA1c, %	8.27 ± 0.87	8.36 ± 0.94	8.03 ± 0.88	0.141
Triglycerides, mmol/L	1.78 (1.28, 3.03)	1.47 (0.96, 2.20)	1.72 (1.33, 2.82)	0.174
Total cholesterol, mmol/L	4.98 ± 1.00	4.82 ± 0.98	4.84 ± 0.94	0.722
LDL-C, mmol/L	3.06 ± 0.83	2.85 ± 0.90	2.93 ± 0.88	0.542
HDL-C, mmol/L	1.26 ± 0.33	1.31 ± 0.37	1.20 ± 0.45	0.047
Alanine aminotransferase, U/L	24.1 (18.4, 34.7)	22.5 (13.6, 37.9)	30.3 (20.3, 44.0)	0.102
Aspartate aminotransferase, U/L	23.0 (18.8, 30.0)	23.0 (16.4, 32.7)	25.2 (20.9, 35.7)	0.173
Alkaline phosphatase, U/L	67.3 (57.8, 81.0)	66.0 (55.1, 86.5)	72.0 (57.2, 79.5)	0.794
Total bilirubin, μmol/L	13.5 ± 5.34	13.1 ± 4.92	12.5 ± 5.48	0.496
Blood urea nitrogen, mmol/L	4.61 ± 1.03	5.02 ± 1.43	5.14 ± 1.43	0.085
Serum creatinine, μmol/L	61.8 ± 13.7	63.9 ± 15.5	68.4 ± 13.5	0.057
Uric acid, μmol/L	308.9 ± 84.5	329.4 ± 92.5	335.7 ± 80.0	0.188
Serum amylase, U/L	51.4 (40.8, 58.1)	51.0 (41.5, 62.5)	52.0 (44.5, 63.8)	0.678
Serum lipase, U/L	56.0 (34.4, 86.3)	70.0 (38.6, 106.6)	88.0 (35.6, 126.2)	0.120
ΔHbA1c, %	−1.48 ± 1.05	−1.25 ± 1.31	−0.93 ± 1.03	0.046

Data are presented as mean ± standard deviation, median (interquartile range) or *n* (%). Δ means the post-treatment level minus its baseline level. Abbreviations: HbA1c, glycated hemoglobin; GDF15, growth differentiation factor 15; LDL-C, low-density lipoprotein cholesterol; HDL-C, high-density lipoprotein cholesterol.

**Table 3:** Univariate linear regression analysis of ΔHbA1c and baseline variables (*n* = 166).

Variables	*β* (95% CI)	*p*-Value	*R* ^2^
Age, year	0.032 (0.015, 0.049)	<0.001	0.076
Sex, male	−0.181 (−0.545, 0.183)	0.328	0.006
Diabetes duration, year	0.033 (−0.004, 0.070)	0.079	0.019
Weight, kg	−0.003 (−0.015, 0.010)	0.672	0.001
Body mass index, kg/m^2^	0.006 (−0.037, 0.050)	0.775	0.001
Heart rate, bpm	0.005 (−0.018, 0.027)	0.689	0.001
Systolic blood pressure, mmHg	0.014 (−0.0003, 0.028)	0.056	0.022
Diastolic blood pressure, mmHg	0.010 (−0.011, 0.032)	0.343	0.005
Fasting blood glucose, mmol/L	−0.043 (−0.144, 0.057)	0.398	0.004
2 h postprandial glucose, mmol/L	0.005 (−0.048, 0.059)	0.845	0.0002
HbA1c, %	−0.679 (−0.846, −0.512)	<0.001	0.282
Triglycerides, mmol/L	0.057 (−0.095, 0.209)	0.461	0.003
Total cholesterol, mmol/L	0.243 (0.062, 0.424)	0.009	0.041
LDL-C, mmol/L	0.246 (0.044, 0.447)	0.017	0.034
HDL-C, mmol/L	0.230 (−0.232, 0.693)	0.327	0.006
Alanine aminotransferase, U/L	−0.002 (−0.009, 0.006)	0.689	0.001
Aspartate aminotransferase, U/L	0.002 (−0.012, 0.017)	0.778	0.0005
Alkaline phosphatase, U/L	0.002 (−0.006, 0.011)	0.553	0.002
Total bilirubin, μmol/L	−0.015 (−0.049, 0.020)	0.404	0.004
Blood urea nitrogen, mmol/L	0.100 (−0.034, 0.235)	0.143	0.013
Serum creatinine, μmol/L	0.0003 (−0.012, 0.013)	0.957	0.0002
Uric acid, μmol/L	−0.0005 (−0.002, 0.002)	0.649	0.001
Serum amylase, U/L	0.001 (−0.008, 0.010)	0.859	0.0002
Serum lipase, U/L	0.002 (−0.002, 0.005)	0.305	0.006
GDF15, pg/mL			0.037
Tertile 1	Reference	—	
Tertile 2	0.226 (−0.196, 0.648)	0.291	
Tertile 3	0.553 (0.115, 0.991)	0.014	

Δ means the post-treatment level minus its baseline level. Abbreviations: HbA1c, glycated hemoglobin; LDL-C, low-density lipoprotein cholesterol; HDL-C, high-density lipoprotein cholesterol; GDF15, growth differentiation factor 15.

## Data Availability

The datasets generated during the current study are available from the corresponding authors upon reasonable request.

## Supplementary Materials

The following supporting information can be downloaded at https://www.mdpi.com/article/10.3390/biomedicines14030572/s1, Figure S1: Scatter plots illustrating the relationship between ΔGDF15 (pg/mL) and Δ serum amylase (U/L) or Δ serum lipase (U/L); Figure S2: Scatter plot illustrating the relationship between baseline total cholesterol (mmol/L) and LDL-C (mmol/L); Figure S3: Scatter plots illustrating the relationship between baseline GDF15 (pg/mL) and ΔHbA1c (%) in the participants >35 years old (A) and ≤35 years old (B); Table S1: Baseline characteristics of the participants included and excluded in this secondary analysis; Table S2: Bidirectional stepwise linear regression analysis of ΔHbA1c for model of baseline GDF15, age, diabetes duration, baseline HbA1c, total cholesterol, systolic blood pressure, and body mass index (*n* = 166); Table S3: Baseline characteristics, ΔHbA1c and ΔGDF15 of the participants between the younger (≤35 years old) and older (>35 years old) adult subgroups; Table S4: Univariate linear regression analysis of ΔHbA1c and baseline variables in the participants >35 years old (*n* = 148); Table S5: Bidirectional stepwise linear regression analysis of ΔHbA1c for model of baseline GDF15, age, diabetes duration, baseline HbA1c, total cholesterol, systolic blood pressure, and body mass index in the participants >35 years old (*n* = 148); Table S6: Linear regression analysis of ΔHbA1c and baseline GDF15 in the participants ≤35 years old (*n* = 18).

## Abbreviations

The following abbreviations are used in this manuscript:

BMI	Body mass index
CI	Confidence interval
FGF21	Fibroblast growth factor 21
GDF15	Growth differentiation factor 15
GLP-1RAs	Glucagon-like peptide-1 receptor agonists
HbA1c	Glycated hemoglobin
HDL-C	High-density lipoprotein cholesterol
LDL-C	Low-density lipoprotein cholesterol
T2DM	Type 2 diabetes mellitus
WHO	World Health Organization
