# Supplementary material for: Circulating GDF15 and HbA1c Response to Add-On Exenatide Therapy in Type 2 Diabetes: A Post Hoc Analysis from a Multicenter Trial
Source: Biomedicines. 2026 Mar 3;14(3):572. doi: 10.3390/biomedicines14030572 (PMC13024465; doi:10.3390/biomedicines14030572)
Supplement: Supplementary file 1 [file biomedicines-14-00572-s001.zip › biomedicines-4111439-supplementary.pdf]

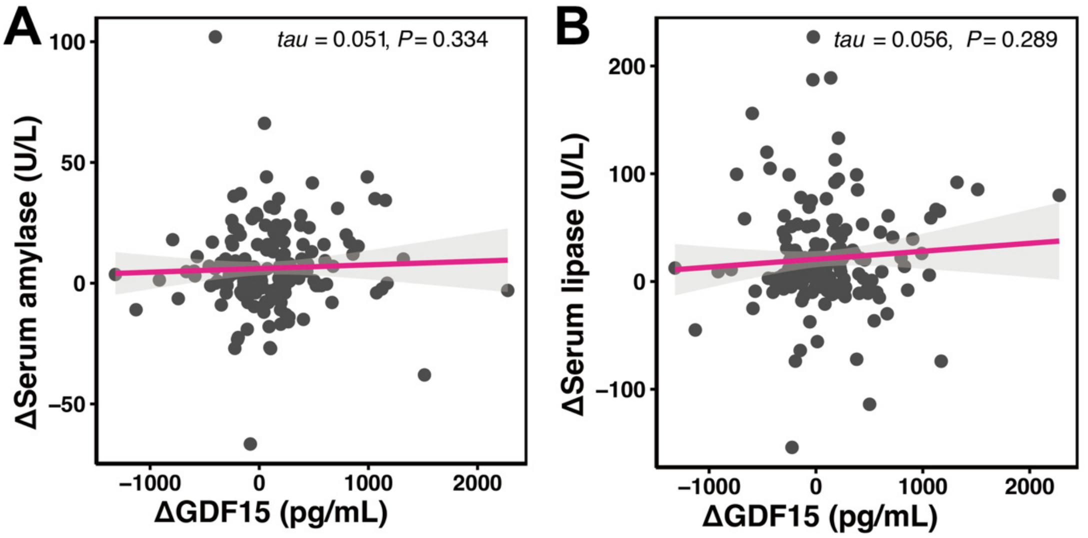

Figure S1. Scatter plots illustrating the relationship between ΔGDF15 (pg/mL) and Δ serum amylase (U/L) or Δ serum lipase (U/L). Correlation coefficients and *p*-Values were assessed by Kendall's *tau* method. Δ means the post-treatment level minus its baseline level. Abbreviation: GDF15, growth differentiation factor 15.

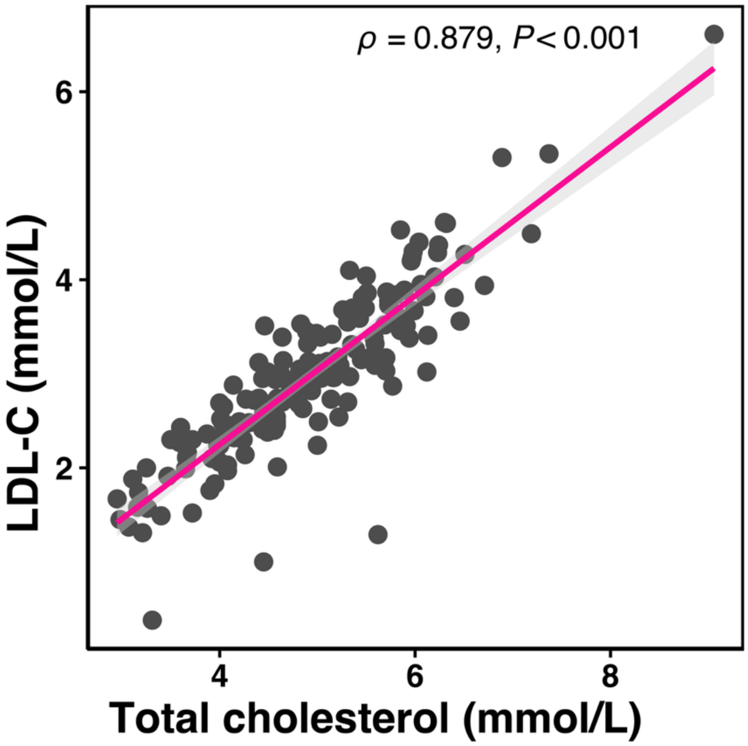

Figure S2. Scatter plot illustrating the relationship between baseline total cholesterol (mmol/L) and LDL-C (mmol/L). Correlation coefficients and *p*-Values were assessed by Spearman's rank method. Abbreviation: LDL-C, low-density lipoprotein cholesterol.

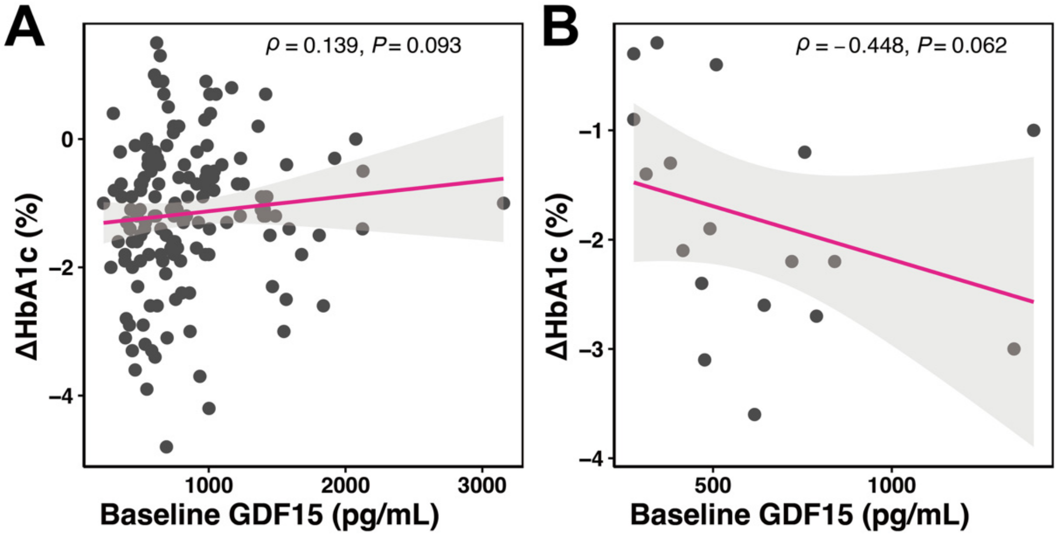

Figure S3. Scatter plots illustrating the relationship between baseline GDF15 (pg/mL) and ΔHbA1c (%) in the participants >35 years old (A) and ≤35 years old (B). Correlation coefficients and *p*-Values were assessed by Spearman's rank method. Δ means the post-treatment level minus its baseline level. Abbreviations: GDF15, growth differentiation factor 15; HbA1c, glycated hemoglobin.

Table S1. Baseline characteristics of the participants included and excluded in this secondary analysis.

**Variables**	**Included** (*n* = 166)	**Excluded** (*n* = 37)	***p*-Value**
Age, year	49.8 ± 10.0	48.1 ± 9.07	0.266
Sex—male, *n* (%)	101 (60.8%)	21 (56.8%)	0.784
Diabetes duration, year	4.79 (2.33, 8.58)	4.58 (2.50, 8.17)	0.768
Weight, kg	80.3 ± 14.7	78.3 ± 16.2	0.255
Body mass index, kg/m^2	28.6 ± 4.14	28.0 ± 4.12	0.466
Heart rate, bpm	76.5 ± 7.90	76.0 ± 8.04	0.690
Systolic blood pressure, mmHg	126.1 ± 12.6	128.0 ± 14.4	0.790
Diastolic blood pressure, mmHg	78.8 ± 8.24	79.1 ± 8.82	0.841
Fasting blood glucose, mmol/L	9.29 ± 1.77	9.94 ± 1.89	0.061
2 h postprandial glucose, mmol/L	16.3 ± 3.34	16.0 ± 4.07	0.693
HbA1c, %	8.23 ± 0.91	8.12 ± 0.89	0.570
Triglycerides, mmol/L	1.67 (1.21, 2.77)	1.58 (1.23, 2.41)	0.941
Total cholesterol, mmol/L	4.88 ± 0.97	4.87 ± 0.98	0.956
LDL-C, mmol/L	2.95 ± 0.87	3.00 ± 0.82	0.716
HDL-C, mmol/L	1.26 ± 0.39	1.20 ± 0.37	0.319
Alanine aminotransferase, U/L	25.6 (17.4, 40.5)	20.0 (14.9, 40.0)	0.363
Aspartate aminotransferase, U/L	24.7 (18.0, 32.2)	27.0 (19.0, 32.0)	0.911
Alkaline phosphatase, U/L	68.3 (57.0, 81.0)	72.0 (62.0, 83.0)	0.283
Total bilirubin, μmol/L	13.0 ± 5.22	12.7 ± 3.69	0.680
Blood urea nitrogen, mmol/L	4.92 ± 1.32	4.87 ± 1.18	0.839
Serum creatinine, μmol/L	64.5 ± 14.5	64.4 ± 15.8	0.971
Uric acid, μmol/L	324.4 ± 86.4	296.0 ± 89.8	0.067
Serum amylase, U/L	51.1 (43.0, 62.0)	47.0 (37.0, 57.0)	0.061
Serum lipase, U/L	64.0 (37.1, 100.8)	48.2 (33.0, 90.0)	0.320

Data are presented as mean ± standard deviation, median (interquartile range), or *n* (%). Abbreviations: HbA1c, glycated hemoglobin; LDL-C, low-density lipoprotein cholesterol; HDL-C, high-density lipoprotein cholesterol

Table S2. Bidirectional stepwise linear regression analysis of ΔHbA1c for model of baseline GDF15, age, diabetes duration, baseline HbA1c, total cholesterol, systolic blood pressure, and body mass index (n = 166).

**Variables**	**Standard *β***	***β* (95% CI)**	***p*-Value**	R^2	**Adjusted** R^2
				0.367	0.351
Age, year	0.161	0.018 (0.002, 0.035)	0.024		
Diabetes duration, year	0.108	0.026 (–0.007, 0.06)	0.124		
Total cholesterol, mmol/L	0.171	0.205 (0.056, 0.354)	0.007		
HbA1c, %	–0.514	–0.658 (–0.819, –0.496)	<0.001		

Δ means the post-treatment level minus its baseline level. Abbreviations: HbA1c, glycated hemoglobin; GDF15, growth differentiation factor 15.

Table S3. Baseline characteristics, ΔHbA1c and ΔGDF15 of the participants between the younger (≤35 years old) and older (>35 years old) adult subgroups.

Variables	Younger (n = 18)	Older (n = 148)	p-Value
Age, year	30.6 ± 2.59	52.1 ± 7.84	<0.001
Sex—male, n (%)	14 (77.8%)	87 (58.8%)	0.134
Diabetes duration, years	2.04 (1.12, 2.38)	5.71 (3.00, 9.71)	<0.001
Weight, kg	92.3 ± 15.3	78.8 ± 14.0	0.002
Body mass index, kg/m^2	31.2 ± 4.61	28.3 ± 3.97	0.009
Heart rate, bpm	77.1 ± 10.2	76.5 ± 7.62	0.796
Systolic blood pressure, mmHg	122.8 ± 12.4	126.5 ± 12.6	0.221
Diastolic blood pressure, mmHg	79.8 ± 7.68	78.7 ± 8.32	0.759
Fasting blood glucose, mmol/L	9.26 ± 1.75	9.29 ± 1.78	0.952
2 h postprandial glucose, mmol/L	14.8 ± 3.26	16.4 ± 3.32	0.066
HbA1c, %	8.43 ± 0.83	8.20 ± 0.92	0.208
Triglycerides, mmol/L	2.21 (1.42, 3.69)	1.65 (1.21, 2.65)	0.075
Total cholesterol, mmol/L	4.97 ± 1.22	4.87 ± 0.94	0.870
LDL-C, mmol/L	3.08 ± 1.09	2.93 ± 0.84	0.860
HDL-C, mmol/L	1.15 ± 0.34	1.27 ± 0.39	0.144
Alanine aminotransferase, U/L	44.4 (34.5, 88.5)	23.1 (16.2, 36.7)	<0.001
Aspartate aminotransferase, U/L	25.1 (21.8, 42.8)	24.2 (18.0, 31.3)	0.274
Alkaline phosphatase, U/L	68.0 (55.8, 77.4)	72.8 ± 21.8	0.587
Total bilirubin, μmol/L	12.7 ± 5.70	13.1 ± 5.18	0.515
Blood urea nitrogen, mmol/L	4.66 ± 1.30	4.95 ± 1.32	0.390
Serum creatinine, μmol/L	64.7 ± 15.2	64.5 ± 14.5	0.974
Uric acid, μmol/L	385.9 ± 110.3	316.9 ± 80.3	0.006
Serum amylase, U/L	46.9 (40.0, 52.5)	52.0 (43.0, 64.0)	0.067
Serum lipase, U/L	62.1 (46.2, 82.5)	64.0 (36.7, 102.3)	0.876
GDF15, pg/mL	500.1 (389.4, 747.4)	743.1 (543.0, 1006.4)	0.009
ΔHbA1c, %	–1.81 ± 1.02	–1.16 ± 1.16	0.020
ΔGDF15, pg/mL	–160.2 (–342.3, 147.4)	76.0 (–141.2, 276.9)	0.020

Data are presented as mean ± standard deviation, median (interquartile range), or n (%). Δ means the post-treatment level minus its baseline level. Abbreviations: HbA1c, glycated hemoglobin; GDF15, growth differentiation factor 15; LDL-C, low-density lipoprotein cholesterol; HDL-C, high-density lipoprotein cholesterol.

Table S4. Univariate linear regression analysis of ΔHbA1c and baseline variables in the participants >35 years old (n = 148).

**Variables**	***β* (95% CI)**	***p*-Value**	R^2
Age, year	0.034 (0.011, 0.058)	0.004	0.055
Sex, male	–0.100 (–0.483, 0.284)	0.609	0.002
Diabetes duration, year	0.023 (–0.016, 0.062)	0.247	0.009
Weight, kg	0.003 (–0.01, 0.017)	0.619	0.002
Body mass index, kg/m^2	0.025 (–0.022, 0.073)	0.293	0.008
Heart rate, bpm	0.013 (–0.012, 0.038)	0.315	0.007
Systolic blood pressure, mmHg	0.016 (0.001, 0.031)	0.037	0.029
Diastolic blood pressure, mmHg	0.014 (–0.009, 0.036)	0.236	0.010
Fasting blood glucose, mmol/L	–0.060 (–0.166, 0.046)	0.264	0.009
2 h postprandial glucose, mmol/L	–0.004 (–0.062, 0.053)	0.876	0.0002
HbA1c, %	–0.674 (–0.850, –0.499)	<0.001	0.284
Triglycerides, mmol/L	0.108 (–0.060, 0.275)	0.207	0.011
Total cholesterol, mmol/L	0.288 (0.092, 0.485)	0.004	0.054
LDL-C, mmol/L	0.295 (0.075, 0.514)	0.009	0.046
HDL-C, mmol/L	0.216 (–0.270, 0.702)	0.381	0.005
Alanine aminotransferase, U/L	0.005 (–0.004, 0.015)	0.257	0.009
Aspartate aminotransferase, U/L	0.013 (–0.005, 0.030)	0.156	0.014
Alkaline phosphatase, U/L	0.003 (–0.006, 0.012)	0.486	0.003
Total bilirubin, μmol/L	–0.011 (–0.048, 0.026)	0.553	0.002
Blood urea nitrogen, mmol/L	0.117 (–0.025, 0.258)	0.107	0.018
Serum creatinine, μmol/L	0.001 (–0.012, 0.015)	0.829	0.0003
Uric acid, μmol/L	0.0003 (–0.002, 0.003)	0.799	0.0004
Serum amylase, U/L	–0.001 (–0.010, 0.009)	0.894	0.0001
Serum lipase, U/L	0.002 (–0.002, 0.005)	0.352	0.006
GDF15, pg/mL			0.037
Tertile 1	Reference	—	
Tertile 2	0.346 (–0.108, 0.799)	0.134	
Tertile 3	0.584 (0.131, 1.038)	0.012	

Δ means the post-treatment level minus its baseline level. Abbreviations: HbA1c, glycated hemoglobin; LDL-C, low-density lipoprotein cholesterol; HDL-C, high-density lipoprotein cholesterol; GDF15, growth differentiation factor 15.

Table S5. Bidirectional stepwise linear regression analysis of ΔHbA1c for model of baseline GDF15, age, diabetes duration, baseline HbA1c, total cholesterol, systolic blood pressure, and body mass index in the participants >35 years old (*n* = 148).

**Variables**	**Standard *β***	***β* (95% CI)**	***p*-Value**	***R*²**	**Adjusted *R*²**
				0.371	0.349
GDF15, pg/mL					
Tertile 1	Reference	Reference	—		
Tertile 2	0.137	0.336 (–0.048, 0.721)	0.086		
Tertile 3	0.156	0.383 (0.002, 0.764)	0.049		
Age, year	0.138	0.020 (0.0001, 0.041)	0.048		
Total cholesterol, mmol/L	0.195	0.242 (0.077, 0.407)	0.004		
HbA1c, %	–0.496	–0.628 (–0.797, –0.459)	<0.001		

Δ means the post-treatment level minus its baseline level. Abbreviations: HbA1c, glycated hemoglobin; GDF15, growth differentiation factor 15.

Table S6. Linear regression analysis of ΔHbA1c and baseline GDF15 in the participants ≤35 years old (*n* = 18).

Variables	Standard *β*	*β* (95% CI)	*p*-Value	R^2	Adjusted R^2
**Model 1: Baseline GDF15**				0.098	0.042
GDF15, pg/mL	−0.313	−0.001 (−0.002, 0.001)	0.206		
**Model 2: Baseline GDF15 and HbA1c**				0.284	0.189
GDF15, pg/mL	−0.256	−0.001 (−0.002, 0.001)	0.264		
HbA1c, %	−0.435	−0.535 (−1.111, 0.042)	0.067		

Δ means the post-treatment level minus its baseline level. Abbreviations: HbA1c, glycated hemoglobin; GDF15, growth differentiation factor 15.
